# Human-Like Neutralizing Antibodies Protect Mice from Aerosol Exposure with Western Equine Encephalitis Virus

**DOI:** 10.3390/v10040147

**Published:** 2018-03-24

**Authors:** Crystal W. Burke, Jeffrey W. Froude, Sebastian Miethe, Birgit Hülseweh, Michael Hust, Pamela J. Glass

**Affiliations:** 1 United States Army Medical Research Institute for Infectious Diseases, Fort Detrick, MD 21702, USA; crystal.w.burke.ctr@mail.mil (C.W.B.); jeffrey.w.froude2.mil@mail.mil (J.W.F.); 2 Technische Universität Braunschweig, Institut für Biochemie, Biotechnologie und Bioinformatik, Spielmannstr.7, 38106 Braunschweig, Germany; miethe.sem@icloud.com (S.M.); m.hust@tu-bs.de (M.H.); 3 Wehrwissenschaftliches Institut für Schutztechnologien (WIS)—ABC-Schutz, Humboldtstr. 1, 29623 Munster, Germany; BirgitHuelseweh@bundeswehr.org; 4 YUMAB GmbH, Science Campus Braunschweig Süd, Inhoffenstr.7, 38124 Braunschweig, Germany

**Keywords:** western equine encephalitis virus (WEEV), alphavirus, monoclonal antibody (mAb), scFv-Fc, NHP antibodies, passive vaccine, aerosol challenge, antibody engineering

## Abstract

Western equine encephalitis virus (WEEV) causes symptoms in humans ranging from mild febrile illness to life-threatening encephalitis, and no human medical countermeasures are licensed. A previous study demonstrated that immune serum from vaccinated mice protected against lethal WEEV infection, suggesting the utility of antibodies for pre- and post-exposure treatment. Here, three neutralizing and one binding human-like monoclonal antibodies were evaluated against WEEV aerosol challenge. Dose-dependent protection was observed with two antibodies administered individually, ToR69-3A2 and ToR68-2C3. In vitro neutralization was not a critical factor for protection in this murine model, as ToR69-3A2 is a strong neutralizing antibody, and ToR68-2C3 is a non-neutralizing antibody. This result highlights the importance of both neutralizing and non-neutralizing antibodies in the protection of mice from WEEV lethality.

## 1. Introduction

The single-stranded positive-sense RNA virus, western equine encephalitis virus (WEEV), is a member of the Togaviridae family [1]. WEEV is a natural chimera resulting from the recombination of eastern equine encephalitis virus (EEEV) and Sindbis virus (SINV) [2]. In humans, WEEV, EEEV and Venezuelan equine encephalitis virus (VEEV) cause disease symptoms ranging from a mild febrile illness to severe encephalitis that may lead to mortality [1]. Disease severity is influenced by multiple factors including viral strain, dose and route of inoculation, as well as health status and age of the patient [1,3,4,5]. WEEV has caused sporadic outbreaks in horses and humans [6,7,8]. Most recently, an outbreak in Uruguay in 2009 resulted in a fatal human case [9]. Disease severity coupled with the ease of production and dissemination of the encephalitic alphaviruses has resulted in these agents designated as Category B pathogens and highlights the need to develop effective medical countermeasures in the event of a biological attack. Other arboviruses, such as chikungunya and Zika virus, have demonstrated rapid shifts in disease prevalence causing widespread epidemics, demonstrating the importance of preparation for potentially emerging infectious diseases.

The pursuit for a vaccine that is safe and efficacious against alphavirus exposure has revealed an essential role of antibodies in the control of virus infection [10,11,12,13]. Furthermore, the level of neutralizing antibody response elicited by a vaccine candidate often determines its continued development. Passive transfer of hyperimmune serum from WEEV-vaccinated animals into naïve recipients was protective [13,14,15], making the use of antibody therapeutics attractive as a rapidly deployable medical countermeasure. For this reason, monoclonal antibodies against other alphaviruses, including VEEV [16] and Semliki Forest virus [17], have been under development since the 1980s. Humanized murine [16,18], nonhuman primate (NHP) [19,20], as well as human [21] antibodies have been developed as potential medical countermeasures.

Previously, four human-like antibodies were identified from immune antibody gene libraries constructed from inactivated WEEV-vaccinated macaques [19]. While all four antibodies bound WEEV antigen by ELISA, only three of the four had varying degrees of WEEV neutralizing activity in an in vitro assay. Here, the ability of these four WEEV-specific monoclonal antibodies to protect mice from a lethal WEEV aerosol exposure was examined.

## 2. Materials and Methods

### 2.1. Ethics Statement and Animal Care

Research was conducted under a USAMRIID Institute Animal Care and Use Committee-approved protocol (9 May 2016) in compliance with the Animal Welfare Act, Public Health Service Policy, and other Federal statutes and regulations relating to animals and experiments involving animals. The USAMRIID is accredited by the Association for Assessment and Accreditation of Laboratory Animal Care, International, and adheres to principles stated in the Guide for the Care and Use of Laboratory Animals, National Research Council, 2011.

### 2.2. Antibody Preparation

The antibodies ToR68-2C3, ToR68-2E9, ToR68-3G2 and ToR69-3A2 were produced as scFv-Fc with a human Fc part as described previously [22].

### 2.3. Virus Stock

The WEEV Fleming stock was obtained from the World Reference Center for Emerging Viruses and Arboviruses (Galveston, TX). The stock was amplified three times in Vero cells from a lyophilized stock that had undergone five passages through suckling mouse brain.

### 2.4. In Vivo WEEV Challenge

Specific pathogen-free, eight-week-old BALB/c mice (*n* = 10/group; Charles River Laboratories) were utilized as a model for WEEV infection. Mice received a single inoculation of monoclonal antibody, irrelevant anti-Marburg virus (100 µg) antibody [23] or PBS intraperitoneally approximately 24 h prior to challenge. Based on previous LD_50_ studies, mice were exposed to a target inhaled dose of 1 × 10^3^ PFU by the aerosol route using the Automated Bioaerosol Exposure System (ABES) II, inside a Class III biological safety cabinet. All mice were weighed on Days 1–14 after challenge and were monitored daily throughout the study for clinical signs of disease.

## 3. Results

### WEEV mAb Prophylaxis in Mice

**Figure 1:**
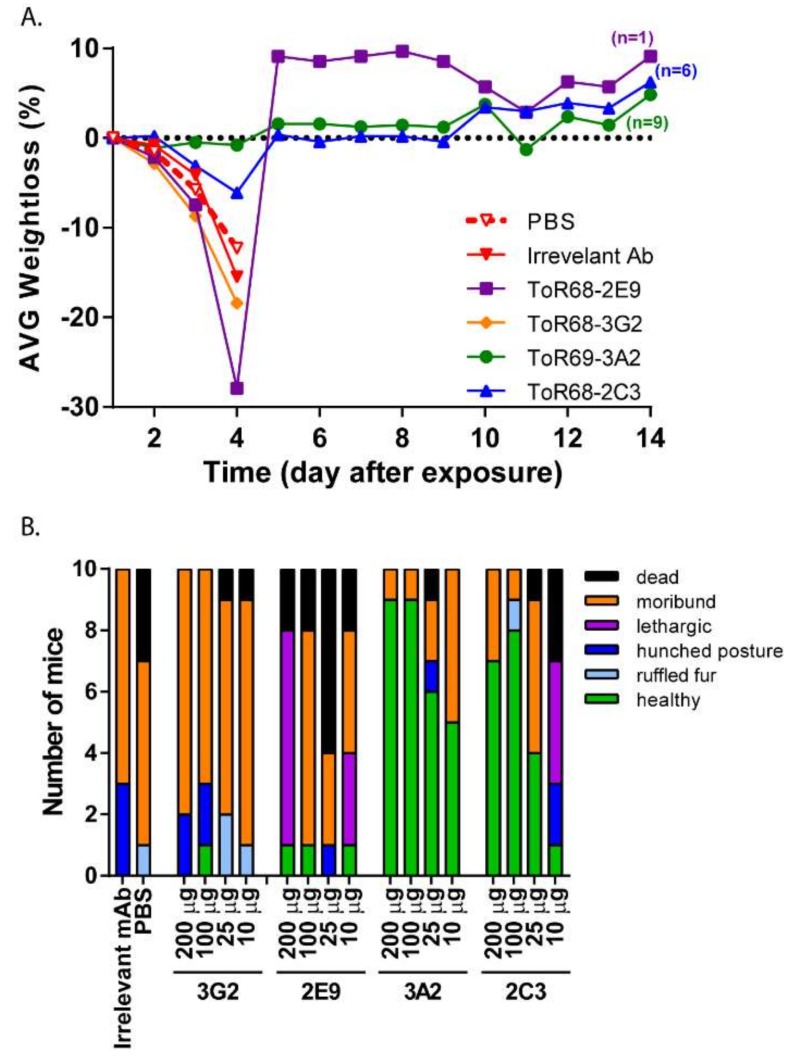
Morbidity of mice treated with anti-western equine encephalitis virus (WEEV) monoclonal antibodies following WEEV exposure. (**A**) Cohorts of 10 mice were administered 200 µg/mouse of ToR68-2E9 (purple), ToR68-2C3 (blue), ToR69-3A2 (green), Tor68-3G2 (orange), irrelevant anti-Marburg virus (100 µg/mouse; solid red) mAb per mouse or an equivalent volume of PBS (dashed red) mAb i.p. 24 h prior to aerosol exposure to WEEV Fleming (10^3^ PFU). Mice were weighed daily, and the percent weight loss was determined by comparison to pre-challenge day weights. (**B**) Clinical observations were made twice daily. Mice were moribund when displaying neurological signs of disease or were unresponsive to stimulus. Data shown are the clinical signs observed on Day 4 (average survival day) post-exposure.

**Figure 2:**
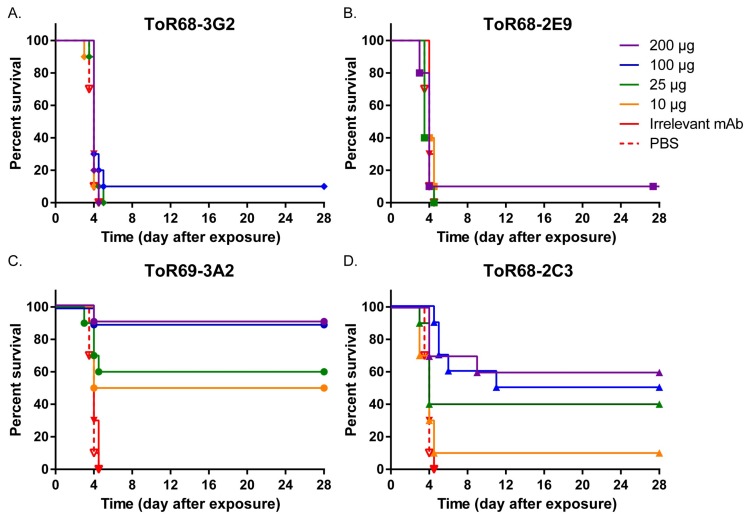
Mortality of mice treated with anti-WEEV monoclonal antibodies following WEEV exposure. (**A**) Mice administered ToR68-3G2. (**B**) Mice administered ToR68-2E9. (**C**) Mice administered ToR69-3A2. (**D**) Mice administered ToR68-2C3. Cohorts of 10 mice were administered 200 µg (purple), 100 µg (blue), 25 µg (green), 10 µg (orange) mAb per mouse, 100 µg irrelevant anti-Marburg virus (solid red) mAb per mouse or an equivalent volume of PBS (dashed red) i.p. 24 h prior to aerosol exposure to WEEV Fleming (10^3^ PFU). Mice were observed twice daily for clinical signs of disease and humanely euthanized when moribund.

ToR68-2C3, ToR68-2E9, ToR68-3G2 and ToR69-3A2 were previously identified to have WEEV binding activity [19]. Additionally, ToR68-2E9, ToR68-3G2 and ToR69-3A2 neutralized WEEV, while ToR68-2C3 did not [19]. To examine the ability of these mAbs to protect mice from WEEV exposure, BALB/c mice (*n* = 10/group) were administered a single-dose of decreasing concentrations (200 µg–10 µg per mouse) of the mAbs by the intraperitoneal (i.p.) route. Approximately 24 h after mAb administration, mice were exposed by the aerosol route to a target inhaled dose of 1 × 10^3^ PFU of the WEEV Fleming strain [24]. Mice were monitored for clinical signs of disease and were euthanized when moribund. As expected, an aerosol challenge with WEEV Fleming resulted in rapid weight loss (Figure 1A) and disease progression with signs of neurological disease including hyper-reactivity and circling (Figure 1B). By five days post-exposure, 100% lethality was observed with an average survival time of four days for mice administered PBS or irrelevant mAb (Figure 2). Two of the neutralizing mAbs, ToR68-3G2 and ToR68-2E9 provided no significant protection from the lethal WEEV aerosol exposure with an average survival time (AST) of four days for all dose groups (Figure 2A,B). A dose-dependent increase in survival was observed for the neutralizing ToR69-3A2 mAb with a 90% survival rate at the 200-µg dose and a 50% survival rate at the lowest dose (10 µg; Figure 2C). Somewhat surprisingly, treatment with the non-neutralizing Tor68-2C3 mAb resulted in 60% survival at the highest dose tested (Figure 2D), and the level of protection observed with this non-neutralizing mAb was dose-dependent. Both ToR69-3A2 and ToR68-2C3 reduced or eliminated clinical signs of disease (Figure 1B).

## 4. Discussion

Anti-WEEV immune serum from rabbits [23], mice [14] and NHPs [25] passively administered to mice can protect against lethal WEE disease. However, to date, no neutralizing or protective murine, human or human-like anti-WEEV monoclonal antibodies have been identified [26]. Here, we demonstrated the first use of a monoclonal antibody prophylaxis to protect mice challenged by a lethal WEEV aerosol exposure. Importantly, protection was observed with a single dose of either a neutralizing antibody ToR69-3A2 or a non-neutralizing antibody ToR68-2C3. Non-neutralizing monoclonal antibodies have demonstrated some success in mouse protection studies against other alphaviruses, including Sindbis virus [27], Semliki Forest virus [17] and VEEV [20,28]. The mechanism of action of ToR68-2C3 is undefined, but protection may be attributed to complement-mediated lysis or antibody-dependent cell-mediated lysis of infected cells [29,30]. Together, these data highlight the importance of in vivo evaluation of both neutralizing and non-neutralizing monoclonal antibodies for protective efficacy.

Despite efforts in antibody identification against alphaviruses, this is the first report of a monoclonal antibody that protected mice against a WEEV challenge. In vaccination studies delivering WEEV, EEEV and VEEV antigen concomitantly, WEEV antigens appear to be less immunogenic in comparison to VEEV and EEEV antigens. This is evidenced by lower neutralizing and binding antibody titers to WEEV vaccine components when similar protein concentrations are delivered in a trivalent formulation [25,31]. Alternatively, the reduced immunogenicity of WEEV antigen in these studies may be a result of immunologic interference similar to what was observed in humans after sequential alphavirus vaccine administration [32,33]. Despite the potential lower WEEV immunogenicity, the vaccines tested protected 100% of mice, suggesting the combination of neutralizing and binding antibodies could be important to confer protection. In comparison to the other encephalitic alphaviruses, WEEV’s reduced protein immunogenicity may contribute to the difficulty of identifying a single WEEV monoclonal antibody that is completely protective.

Neither antibody provided 100% protection from lethality after WEEV aerosol exposure even at the 200 µg dose. Studies with passive administration of immune serum from NHPs [25,34] and transchromosomic bovine IgG [35] into mice have found that a single administration was not 100% protective against a high dose aerosol exposure of VEEV [35], EEEV or WEEV [25,34]; however, delivery of a second administration resulted in 90–100% survival. This would suggest that a second administration of ToR69-3A2 or ToR68-2C3 may result in enhanced survival. Another possibility is the generation of antibody-escape mutants, which have been identified for other viruses including Ebola virus [36], Dengue virus [37] and influenza virus [38], as well as alphaviruses [39,40]. Therefore, future studies will evaluate this possibility and also the efficacy of a cocktail of ToR69-3A2 and ToR68-2C3 for enhanced survival benefits.

To summarize, this manuscript describes the first monoclonal antibodies that are protective against WEEV in a mouse in vivo aerosol challenge model.
